# Subacute Massive Pulmonary Embolism Treated With Streptokinase

**DOI:** 10.7759/cureus.11157

**Published:** 2020-10-25

**Authors:** Naisargee N Solanki, Narendra Tanwar, Nilesh D Solanki

**Affiliations:** 1 Medicine, Naisargee Hospital, Bardoli, IND; 2 Cardiology, Maitreya Multisuperspeciality Hospital, Surat, IND

**Keywords:** subacute massive pulmonary embolism, dyspnea, systemic thrombolysis

## Abstract

Subacute massive pulmonary embolism occurs insidiously over weeks, has a high mortality rate, and may be less amenable to systemic thrombolysis. It is associated with a high likelihood of the development of pulmonary hypertension. The subacute presentation makes it difficult to diagnose leading to treatment delays and poor clinical outcomes. We present a case of a 40-year-old man with unprovoked pulmonary embolism and no evidence of deep vein thrombosis. The patient underwent systemic thrombolysis with streptokinase and was given long-term oral anticoagulants. A dramatic clinical recovery was seen along with significant clearance of the thrombus.

## Introduction

Pulmonary embolic disease has a broad array of clinical presentations. Herein we discuss a specific subgroup being subacute massive pulmonary embolism. Subacute massive pulmonary embolism is defined as symptom onset of 2-12 weeks, without a definite episode of cardiovascular collapse and pulmonary angiogram showing massive pulmonary embolus (>50% obstruction in major pulmonary arteries). The most common presentation is progressive dyspnea, pleuritic chest pain, and hemoptysis [1]. Patients presenting sub-acutely have a higher mortality rate and an increased incidence of thromboembolic hypertension compared to patients with an acute presentation [2]. Due to the subacute onset, the diagnosis and treatment are often delayed leading to poor clinical outcomes. Although pulmonary embolism frequently presents with deep vein thrombosis, we present a case of unprovoked isolated pulmonary embolism [3]. This study aims to provide a detailed clinical description of a case of subacute massive pulmonary embolism so that it is more readily recognized and treated.

## Case presentation

**Figure 1:**
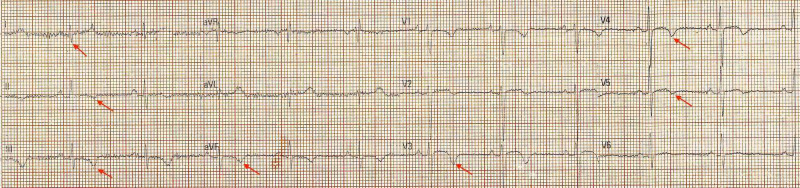
Electrocardiogram showing S wave in Lead I and T wave inversion in Lead II, III, aVF, V3, V4, and V5.

**Figure 2:**
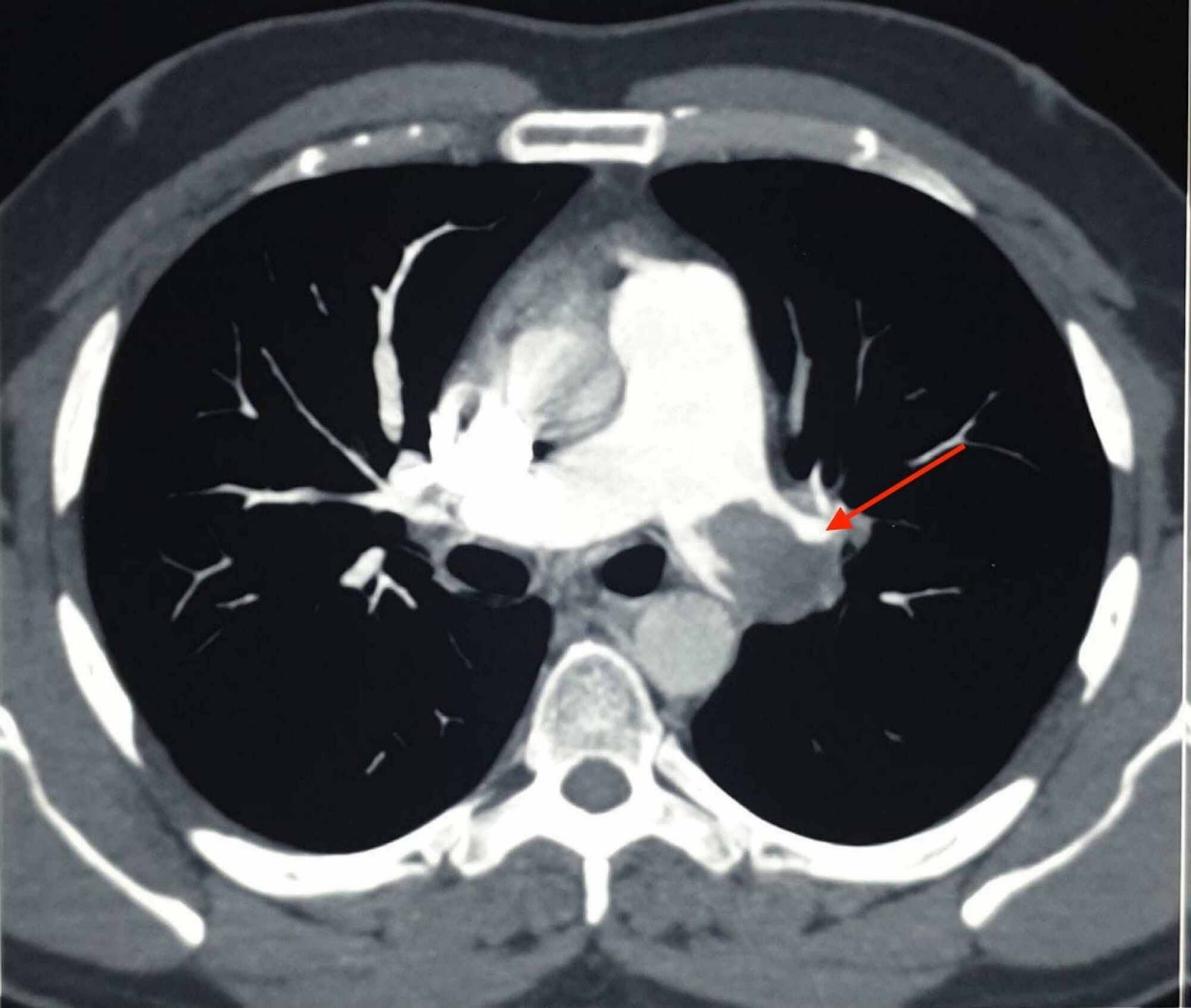
Axial section of CTPA scan showing a large hypodense filling defect in the left main pulmonary artery. CTPA - Computed tomography pulmonary angiogram

A 40-year-old man presented to the emergency department with the chief complaints of uneasiness in the chest, breathlessness, and dyspnea on exertion for 1.5 months. His symptoms exacerbate on climbing stairs and lifting heavy objects. No co-morbid conditions were noted. He is a current smoker and drinks alcohol socially. On general examination his pulse - 66/min, blood pressure (BP) - 130/90 mm of Hg, respiratory rate (RR) - 18/min, jugular venous pressure (JVP) - normal, peripheral capillary oxygen saturation (SpO2) - 98%. The cardiac, respiratory, and abdominal examinations were unremarkable. Electrocardiogram (ECG) was performed which showed S wave in Lead I and T wave inversion in Lead II, III, aVF, V3, V4, V5 (Figure 1). The chest X-ray appeared normal. Initial laboratory findings are noted in Table 1. As the classic retrosternal chest pain was not present and the serum troponin T level was normal, 2-D colour Doppler echocardiography was performed. It showed that the right atrium and right ventricle were dilated, left ventricular ejection fraction (LVEF) was 60%, moderate to severe tricuspid regurgitation (TR) was present, right ventricular systolic pressure (RVSP) was 70 mm of Hg, inferior vena cava (IVC) was dilated (2.5 cm) and non-collapsing and no regional wall motion abnormality (RWMA) was seen at rest. He was further investigated by computed tomography pulmonary angiogram (CTPA) which showed a large hypo-dense defect involving the left main pulmonary artery extending into the left anterior segmental artery and apical branches of the left upper lobar and left inferior lobar artery along with an eccentric hypotenuse filling defect involving the right inferior lobar artery (Figure 2). Hence, subacute massive pulmonary embolism was confirmed.

**Table 1:** Laboratory findings. RBC - Red blood cells; WBC - White blood cells; INR - International normalized ratio; ESR - Erythrocyte sedimentation rate; CPK-Total - Creatine phosphokinase total; CPK-MB - Creatine phosphokinase myocardial band; SGPT - Serum glutamic-pyruvic transaminase; SGOT - Serum glutamic-oxaloacetic transaminase; T3 - Triiodothyronine; T4 - Thyroxine; TSH - Thyroid stimulating hormone.

Parameters	Patient's Values	Reference Values
Hemoglobin	17.1 gm%	13-17 gm%
Total RBC	5.21 million/c.mm	4.5-5.5 million/c.mm
Packed Cell Volume	49.7%	40-50%
Mean Corpuscular Volume	95.4 fL	83-101 fL
Mean Corpuscular Hemoglobin	32.8 pg	27-32 pg
Mean Corpuscular Hemoglobin Concentration	34.4 gm/dL	31.5-34.5 gm/dL
Red Cell Distribution Width	18.9%	11-16%
Total WBC	11,100/µL	4,000-10,000/µL
Platelet Count	233,000/µL	150,000-500,000/µL
Prothrombin time	14.6 seconds	10.3-12.8 seconds
INR	1.17	0.85-1.15
Troponin - T	<0.01 ng/mL	0.0-0.013 ng/mL
ESR	5 mm/after 1 hour	3-5 mm/after 1 hour
C-Reactive Protein	179.9 mg/L	0-5 mg/L
D-dimer	445 ng FEU/mL	0-500 ng FEU/mL
Homocysteine	15.18 µmol/L	1.00-15.39 µmol/L
CPK-Total	114.6 U/L	38-308 U/L
CPK-MB	25.9 U/L	0-25.0 U/L
Serum creatinine	1.06 mg/dL	0.50-1.50 mg/dL
SGPT	52.5 U/L	0-41 U/L
SGOT	24.42 U/L	0-37 U/L
Serum Sodium	142 mEQ/L	136-145 mEQ/L
Serum Potassium	4.65 mEQ/L	3.50-5.10 mEQ/L
Serum Chloride	102 mEQ/L	98-107 mEQ/L
Random Blood Glucose	80.1 mg/dL	70-160 mg/dL
T3	76.63 ng/dL	70-204 ng/dL
T4	7.5 µg/dL	4.6-10.5 µg/dL
TSH	1.2253 µIU/mL	0.4-4.2 µIU/mL

The patient was admitted to the intensive care unit and was started on Streptokinase 250,000 IU IV diluted over 30 minutes then 100,000 IU/hr infusion for 24 hours. The treatment was then shifted to IV heparin 3000 IU 8 hourly for three days along with oral dabigatran, aspirin, clopidogrel, rosuvastatin, sildenafil citrate, and ivabradine. A venous Doppler study of both lower limbs was performed which showed normal venous flow without Doppler evidence of deep vein thrombosis. After clinical recovery, the patient was discharged with oral medications - dabigatran, sildenafil citrate, aspirin (75 mg), and diuretics. On advice, the patient quit smoking and alcohol intake.

**Figure 3:**
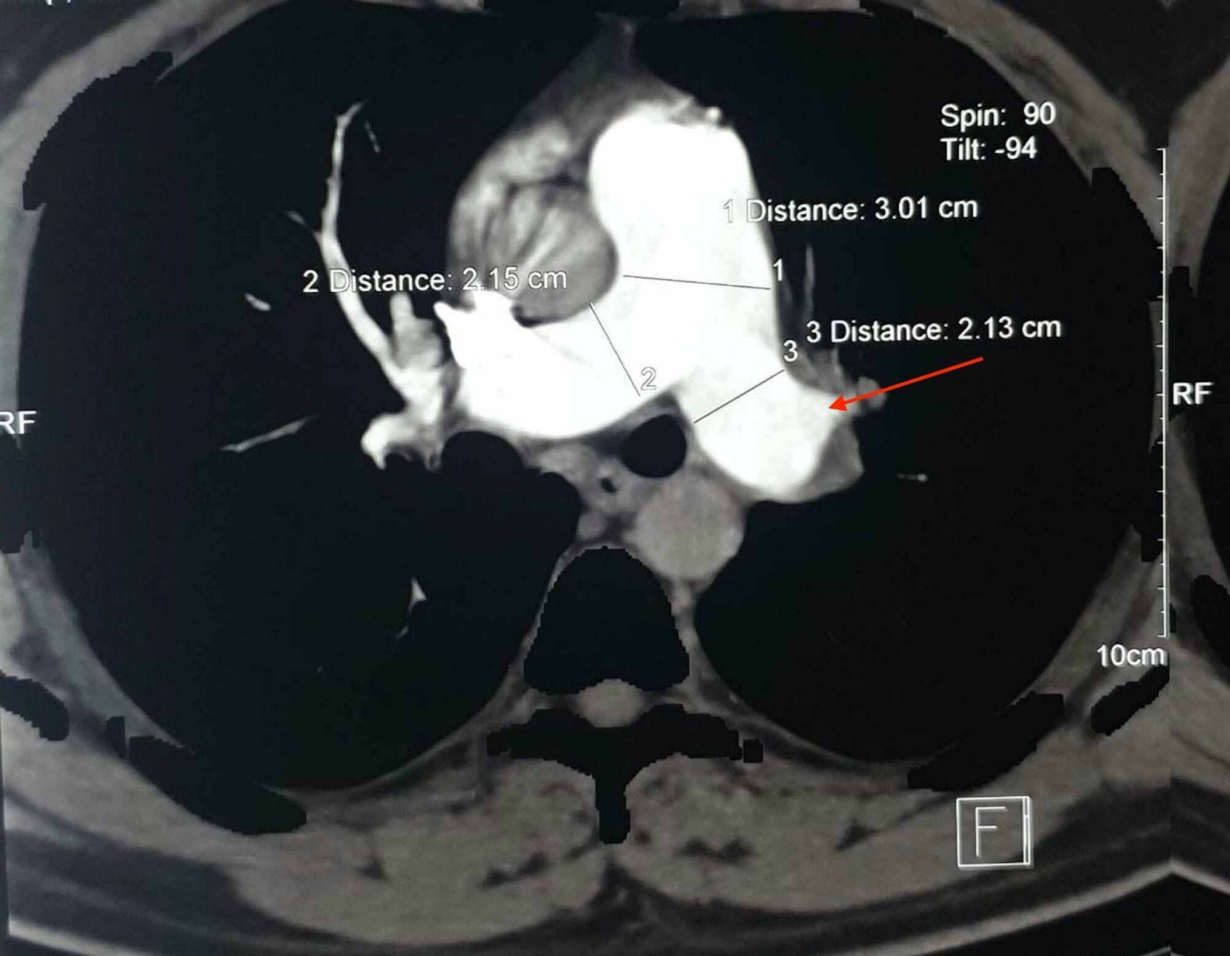
Axial section of CTPA scan showing reduction in the size of the filling defect in left main pulmonary artery. CTPA - Computed tomography pulmonary angiogram

**Figure 4:**
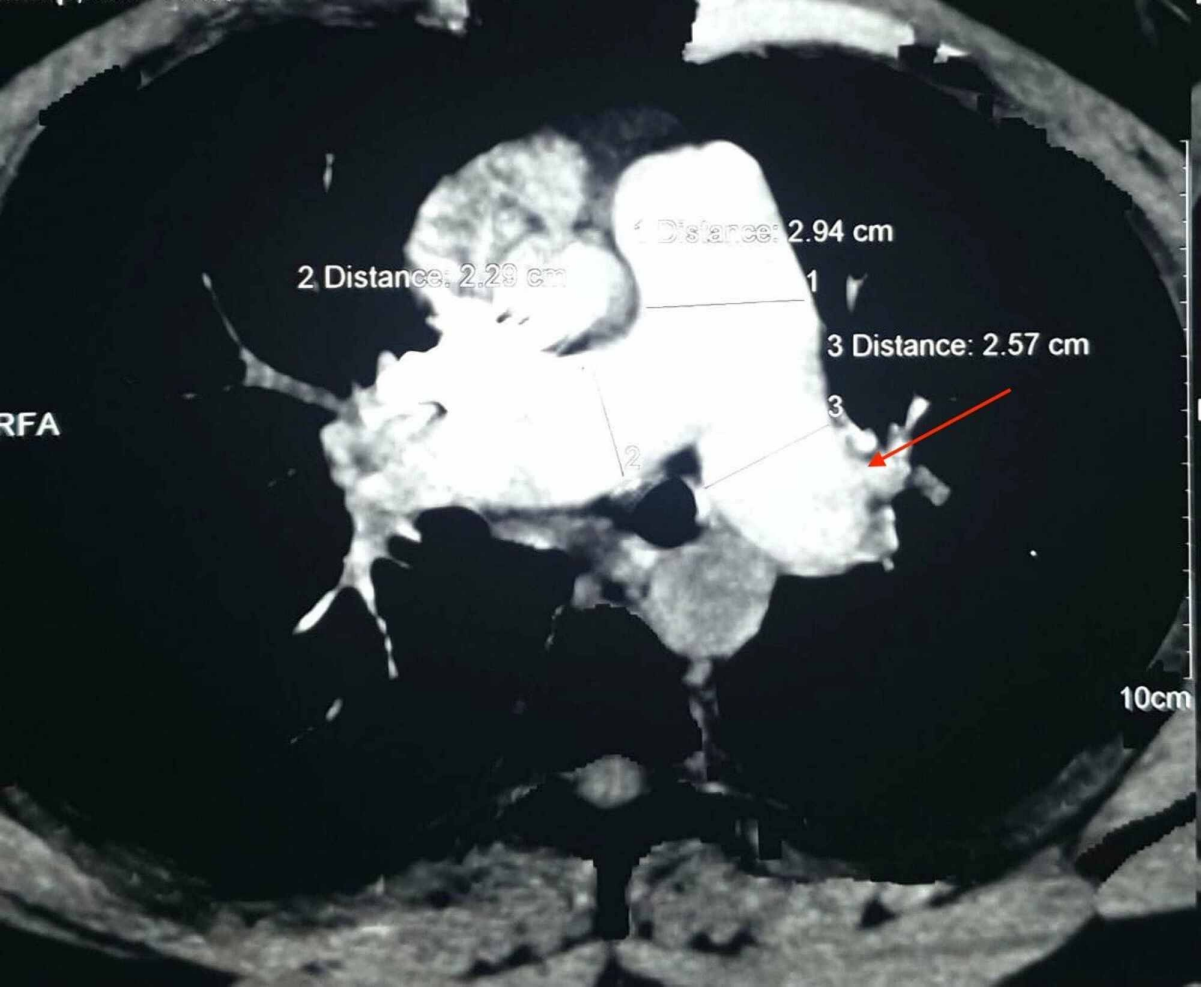
Axial section of CTPA showing minimal reduction in size and extent of filling defect in the left main pulmonary artery compared to previous CTPA scan. CTPA - Computed tomography pulmonary angiogram

The patient came for regular follow-up and was clinically stable without any complaint. Repeat CT pulmonary angiography at the one-month follow-up showed a significant reduction in the extent of thromboembolism but chronic partially occlusive thrombus was still noted (Figure 3). Repeat 2D echocardiography at three months showed pulmonary artery hypertension (PAH) of 35-40 mm of Hg. The patient was advised for pulmonary thromboendarterectomy due to presence of chronic thrombus and pulmonary artery hypertension but he refused to undergo surgery. Repeat CT pulmonary angiography at seven months of follow-up showed a minimal reduction in the extent of thromboembolism compared to the previous CT scan (Figure 4). The patient is currently being managed medically and is clinically stable with no chief complaints.

## Discussion

Pulmonary embolic disease has varied clinical presentations and is classified into subgroups for a better understanding of its clinical course, treatment interventions, and prognosis [2]. The subacute pulmonary embolism commonly presents with progressive dyspnea, pleuritic chest pain, and hemoptysis with the absence of cardiovascular collapse [1]. Its clinical presentation can be similar to acute coronary syndrome which can lead to misdiagnosis and treatment delays [4]. In our case, the ECG showed changes suggestive of the acute coronary syndrome but due to the absence of retrosternal chest pain, co-morbidities, and a normal troponin T level, the patient was further investigated for pulmonary embolism.

The predisposing factor for subacute pulmonary embolism is often unknown and can potentially contribute to recurrent emboli even after initial treatment is started [4-5], but in our case, no recurrent embolism has been noted during 22 months of regular follow-up. The patient currently receives long-term oral anticoagulants as a part of his medical management and this could be the potential cause of the nondevelopment of recurrent emboli. Subacute pulmonary embolism shows a poor response to early streptokinase therapy compared to acute pulmonary embolism possibly as the old thrombus contains less plasminogen than a recent thrombus [5]. In our case, the patient showed dramatic clinical improvement and significant clearance of thrombus in the follow-up CT pulmonary angiogram.

The patient was started on dabigatran - a direct thrombin inhibitor which has been shown to be as effective and safe as the vitamin K antagonist in the treatment of subacute pulmonary embolism and it has also been shown to cause less bleeding complications [6]. The patients who survive a single subacute massive pulmonary embolism have been shown to have a better prognosis compared to patients with discrete repeated episodes of occult embolism [7]. There may be minor persistent pulmonary vasculature abnormalities but these are not associated with clinical disability [5].

## Conclusions

In conclusion, subacute massive pulmonary embolism requires a high level of clinical suspicion and prompt treatment. It is often diagnosed late and has a high mortality rate. Hence, it is imperative to include it in the differential when presented with progressive dyspnea in a patient where the diagnosis is unclear. This case report suggests that systemic thrombolysis along with long-term anticoagulation could be useful in subacute massive pulmonary embolism, but additional studies need to be performed to further define its use.
